# Analysis of Rare Alleles of *miRNA-146a (rs2910164)* and *miRNA-34b/c (rs4938723)* as a Prognostic Marker in Thyroid Cancer in Pakistani Population

**DOI:** 10.3390/diagnostics12102495

**Published:** 2022-10-15

**Authors:** Rashida Khan, Samina Asghar Abbasi, Qaisar Mansoor, Mehvish Naseer Ahmed, Kahkashan Bashir Mir, Ruqia Mehmood Baig

**Affiliations:** 1 Department of Zoology, PMAS-Arid Agriculture University, Rawalpindi 46300, Pakistan; 2 Institute of Biomedical and Genetic Engineering (IBGE), Islamabad 44000, Pakistan; 3 Nuclear Medicine, Oncology and Radiotherapy Institute (NORI), Islamabad 44000, Pakistan

**Keywords:** papillary thyroid carcinoma, *rs4938723*, *miRNA-34b/c*, *miRNA-146a*, *rs2910164*, thyroid cancer

## Abstract

Background: Rationale: The *miRNAs* are short non-coding functional RNAs that are involved in the regulation of transcriptomes. It was found that human *miRNA-146a* and *miRNA34b/c* are important *microRNAs* and are functioning either as *onco-miRNAs*, or acting as tumor suppressors, in different conditions. To date, no study has been performed to evaluate the alterations of *miRNA-146a* *rs2910164* and *miRNA34b/c* *rs4938723* polymorphism as a risk factor in the development of thyroid cancer in the Pakistani population. Mutational analysis of *rs2910164* and *rs4938723* of *miRNA-146a* and *miRNA-34b/c* was carried out to check their association with the development of thyroid carcinogenesis. Material and Methods: Papillary thyroid cancer (PTC) patients with age and gender-matched controls were recruited for the present study. DNA extraction, genotyping of *rs2910164* and *rs4938723* was carried out by ARMS-PCR. Statistical analyses were carried out using SPSS software (version 20). Results: The odds ratio for risk allele C of *rs2910164* for patients and controls was 23.0168 (3.0321–174.7208) with a *p*-value of <0.0001, showing that the frequency of the major allele G was lower in patients while the frequency of minor allele C was higher in patients. Similarly, the odds ratio for risk allele C of *rs4938723* was 1.8621 (1.0321–3.3596) with a *p*-value of <0.03788 showing significant association with the development of thyroid cancer. Conclusions: The study highlights the significant association of miRNAs SNPs as one of the genetic risk factor for PTC. It was concluded that *miRNA-146a (rs2910164)* showed higher frequency of minor allele C in patients. Similarly in *miRNA-34b/c* gene SNP *rs4938723* was observed to have a strong association with the development of thyroid cancer as the frequency of rare allele C was higher in patients.

## 1. Introduction

One of the endocrine related malignancies of the thyroid is papillary thyroid carcinoma (PTC) which has been reportedly observed at an alarming rate across the world populations. Differentiated thyroid carcinoma constitutes about 90% of thyroid cancers, includes papillary thyroid cancer (PTC) and follicular thyroid cancer (FTC) [1]. Papillary thyroid carcinoma (PTC) is the most common type of thyroid cancer that accounts for almost 85–90% of cases [2]. miRNAs (microRNAs) are found endogenously and are single-stranded noncoding 18- to 25-nucleotide RNAs which play a role in various biological and pathological processes of cell proliferation, differentiation, and apoptosis [3]. miRNAs contribute to the development of cancer in two ways. First, there may be the up-regulation of some miRNAs which results in the silencing of tumor suppressor genes. Secondly, there may be the downregulation of miRNAs which could result in more expression of oncogenes. The deregulations of miRNAs may thus lead to the overexpression of some oncogenes or decreased expression of tumor suppressor genes leading to cancer [4]. It is found that aberrations of *miRNA-146* are associated with human thyroid cancer. *miRNA-146a* shares an equivalent seed sequence with *miRNA-146b*, but it is encoded by a special chromosome within the genome [5]. *miRNA-146a* has been shown to contribute to the complex molecular mechanisms involved in the control of cell growth, differentiation, and survival processes primarily associated with cancer development and progression [6]. Both isoforms of *miRNA-146a* and *b* potentially regulate the SMAD4 gene which is a transcription factor that plays a key role in growth and differentiation [7]. The sequence variation of *miRNA-146a* affects the expression of mature miRNA and is linked to the danger of thyroid carcinogenesis [8]. Pre-miRNA-146a C/G polymorphism, designated *rs2910164*, is encoded on chromosome 5q33 and located within the precursor stem region [9].

This important miRNA Single Nucleotide Polymorphism SNP has been studied in PTC and demonstrated to be related to the spread of cancers like prostatic adenocarcinoma and gastric cancer [10]. Jazdzewski et al. (2009) first demonstrated that *rs2910164 (miRNA-146a)* played a key role in genetic predisposition to PTC through regulation of different other miRNAs [8], but later in other studies carried out on Asian or European populations, it was demonstrated that no association is found between the SNP of this miRNA and thyroid cancer. However, the studies carried out by Wei et al. predicted that the *rs2910164* is playing a key role in transferring the benign nodules into PTCs [11]. It was found that miRNAs have many targets and have the potential to fine-tune gene expression and different physiological and pathological processes which initiate the cancer [12]. This study aimed to understand the possible association of this SNP with PTC in the Pakistani population. For this purpose, mutational analysis was done to see the possible risk association of *rs2910164* with PTC.

Another aspect of this study was the mutational analysis of *rs4938723* of *miRNA-34b/c*. Many pieces of evidence have shown that *miRNA-34b/c* plays a role in the development of different types of cancer. It was found that in the development of thyroid cancer *miRNA-34b/c* has an altered expression and contributes to carcinogenesis [3]. It was demonstrated that a change in the CpG island of the promoter region of *pri-miRNA-34b/c* is the reason for the development of cancer. It was revealed that the variation of *rs4938723* C to T may affect the binding of the GATA-X transcription factor which then causes the altered expressions and results in the development of carcinomas [13]. p53 is the main regulator of miRNAs especially the family of *miRNA-34* (i.e., *miR-34a, miR-34b, and miR-34c*), and two different types of primary miRNAs are encoding these three miRNAs. *Pri-miRNA-34b/c* encodes the transcripts of *miRNA-34b* and *miRNA-34c* while *miRNA-34a* is encoded by its own transcript [14].

The p53 is considered as the “guardian of the genome” which shows how much this gene is important in cell cycle control, apoptosis, and maintenance of DNA integrity [15]. It was reported that both p53 and *miRNA-34b/c* have a major role in the development of tumors and alterations of these two have a connection with alterations of *miRNA-34b/c rs4938723* that result in the development of thyroid cancer [16]. To date, no study has been performed to check the alterations of *miRNA34b/c* *rs4938723* polymorphism as a risk factor in the development of thyroid cancer in Pakistan. For this purpose, mutational analysis was carried out to check its association with thyroid carcinogenesis in the Pakistani population.

An association study has reported that the *miR-34b/c rs4938723* CT/CC genotypes were associated with a significantly high risk of hepatocellular cancer [17]. Results resembling this finding were also observed in nasopharyngeal carcinoma, osteosarcoma, and renal cell cancer [18]. Conflicting results also suggest that the variations have different effects on different types of cancers [19]. Gao et al. found that the *miRNA-34b/c rs4938723* CC genotype decreased the risk of colorectal cancer in a Chinese population at about 0.56-fold [20]. In the current study, it was investigated whether either mutational or expressional deregulation of these miRNAs plays a role in the development of PTC and whether these miRNAs can be further investigated as diagnostic and prognostic biomarkers for the early detection of thyroid carcinogenesis in the studied population.

## 2. Material and Methods

### 2.1. Collection of Blood Samples with Their Demographical Data

Two hundred and five confirmed papillary thyroid cancer (PTC) patients with age and gender-matched normal controls were selected for the current study. Demographical and clinical data of the study subjects were obtained on a structured questionnaire. Blood samples were obtained and collected in EDTA coated tubes and stored at low temperature (4 °C). Informed consent statements were signed by patients and healthy individuals. The study protocol was approved by the ethical review committee of PMAS Arid Agriculture University, Rawalpindi and from the hospitals of Rawalpindi and Islamabad, Pakistan (3 October 2018).

### 2.2. Inclusion Criteria/Exclusion Criteria

All those patients who were confirmed as thyroid cancer patients by the oncologists were included in the study while all patients who were having other problems of the neck or mentioned as hypothyroid or hyperthyroid patients were excluded from the study.

### 2.3. Genotyping

The experiments were carried out using tetra ARMS-PCR. For mutational analysis of *rs2910164* of *miRNA-146a* primers were designed by primer blast. Selected primers were forward primers, F-inner-5′-CATGGGTTGTGTCAGTGTCAGACGTG-3′, forward-outer F outer-5′-TAGACCTGGTACTAGGAAGCAGCTGCAT-3′-F and reverse primers R-inner-5′-GATATCCCAGCTGAAGAACTGAATTTGAG-3′reverse outer R-outer-5′-ATACCTTCAGAGCCTGAGACTCTGCCTT-3′-R. Primers were also designed for the selected sequence of DNA for miRNA-34b/c rs4938723 by primer blast. For this purpose, the following forward and reverse primers were designed. Forward inner F-inner-5′-CCTCTGGGAACCTTCTTTGACCTCTC-3′-F, F-outer-5′-CTCCCAGAAGTCCTCTGTAACTGTCCCT-3′-F and reverse primer, R-inner-5′-AGAAGGGAGGTCCTCAATGAGAGCTTTA-3′-R, R-outer-5′-TAGTCAAATAGTGAGCCAGGCAGCTTGT-3′-R were designed for *miRNA-34b/c rs4938723.*

PCR was carried out in a total volume of 25 μL having 5 μL of genomic DNA (20 ng), 2.5 μL 10× PCR buffer (NH4)2SO4, 1 μL of 20 nM forward and reverse primers each, 0.3 μL of 5 U/μL *Taq polymerase* enzyme, 1 μL of 25 mM MgCl_2_, 1 μL of 2 mM dNTPs (Thermo Fisher, Waltham, Massachusetts USA) and PCR water 12.7 μL/sample. The PCR product was analyzed by 2% *w/v* agarose gel electrophoresis. The statistical analysis of data was carried out by SPSS (version 20). *p*-value < 0.05 was considered statistically significant for the T-test. Graphs were constructed by using graph pad prism software.

## 3. Results

### 3.1. Demographical and Clinical Data

The patients were categorized based on their age and gender into different groups. Most of the patients in the present study were females having a frequency of about 86% while males were only 14%. Patients were categorized into three different age groups, i.e., later age (more than 60), middle-age (40–60), and early age (below 40). It was seen that all the patients were having papillary thyroid cancer with its different subtypes (Table 1).

### 3.2. Genotype Frequency Distribution of rs2910164 and rs4938723 with the Risk of Thyroid Cancer in Pakistani Population

Association of *rs2910164* SNP of *miRNA-146a* with the risk of thyroid cancer progression was determined with the help of tetra ARMS-PCR. Hardy–Weinberg calculations for patients’ samples showed that the observed frequencies of homozygous wild type GG, heterozygous CG, and homozygous recessive CC were 60 percent, 38.5 percent, 1.5 percent while the expected frequencies are 62.8056%, 32.888% and 4.3056%, respectively. The observed frequencies of GG, CG, and CC in controls were 98%, 2% and zero% while the expected frequencies were 98.0%, 1.98% and 0.01% respectively. The *p*-value for the difference between the observed and expected frequencies in patients was 0.95, (>0.05) and is statistically non-significant, indicating that there is no significant difference between the expected and observed frequencies of genotypes in the patients. The Chi-square value was 2.9109 for patients. The *p*-value for controls was 0.69 (>0.05), which is also statistically non-significant, indicating no significant difference of observed and expected frequencies in the control, with a Chi-square value of 0.012. It was seen that the rare genotype CC is significantly associated with the risk of development of thyroid cancer with a *p*-value 0.00001 (<0.05) (Table 2).

The relationship of the SNP of *miRNA-34b/c* with the risk of thyroid cancer development was also determined with the help of tetra ARMS-PCR. Hardy–Weinberg calculations for patient samples showed that the observed frequencies of homozygous wild type TT, heterozygous TC, and homozygous recessive CC were 10 percent, 36 percent and 54 percent while the expected frequencies are 8.1225%, 40.755%, and 51.1225%, respectively. The observed frequencies of TT, TC, and CC in controls were 30%, 54% and 15% while the expected frequencies were 32.8182%, 48.3636% and 17.8182%, respectively. The *p*-value for the difference between the observed and expected frequencies in patients was 0.654129 (>0.05), and is statistically non-significant, indicating that there is no significant difference between the expected and observed frequencies of genotypes in patients. The Chi-square value was =0.8489 for patients. The *p*-value for controls was 0.510533 (>0.05), which is also statistically non-significant indicating no significant difference of observed and expected frequencies in the control; the Chi-square value was 1.3446. It was also observed that the rare genotype CC is significantly associated with the risk of development of thyroid cancer with a *p*-value 2 × 10^−8^ (<0.05) (Table 2).

#### 3.2.1. Combined Genotype Frequency Effect in *miRNA-146a rs2910164* and *miRNA-34b/c (rs4938723)*

The combined effect of genotype frequencies in patients and control group was calculated for *rs2910164*. The frequency of homozygous dominant GG was 60% and 98% in patients and controls, respectively, while the combined frequency of heterozygous and homozygous mutant CG/CC was 40% and 2% in patients and controls, respectively. The *p*-value for the genotypes frequency in patients and control was 0.00001 (<0.05), with a chi-square value of 43.52. The *p*-value was statistically significant (<0.05) indicating that the combined effect of genotypes and allele frequency showed statistically significant involvement with the development of disease. The combined effect of genotype frequencies in patients and the control group was also calculated for *rs4938723*. The frequency of homozygous dominant TT was 10% and 30% in patients and controls, respectively, while the combined frequency of heterozygous and homozygous mutant TC/CC was 90% and 68% in patients and controls, respectively. The *p*-value for the genotypes frequency in patients and control was 0.00030426 (<0.05), with a chi-square value of 13.044. The *p*-value was statistically significant (<0.05) indicating that the combined effect of genotypes and allele frequencies are significantly involved in the development of disease (Table 3).

#### 3.2.2. Odds Ratios Calculations for Genotypes of *miRNA-146a rs2910164* and *miRNA-34b/c rs4938723*

**Figure 1:**
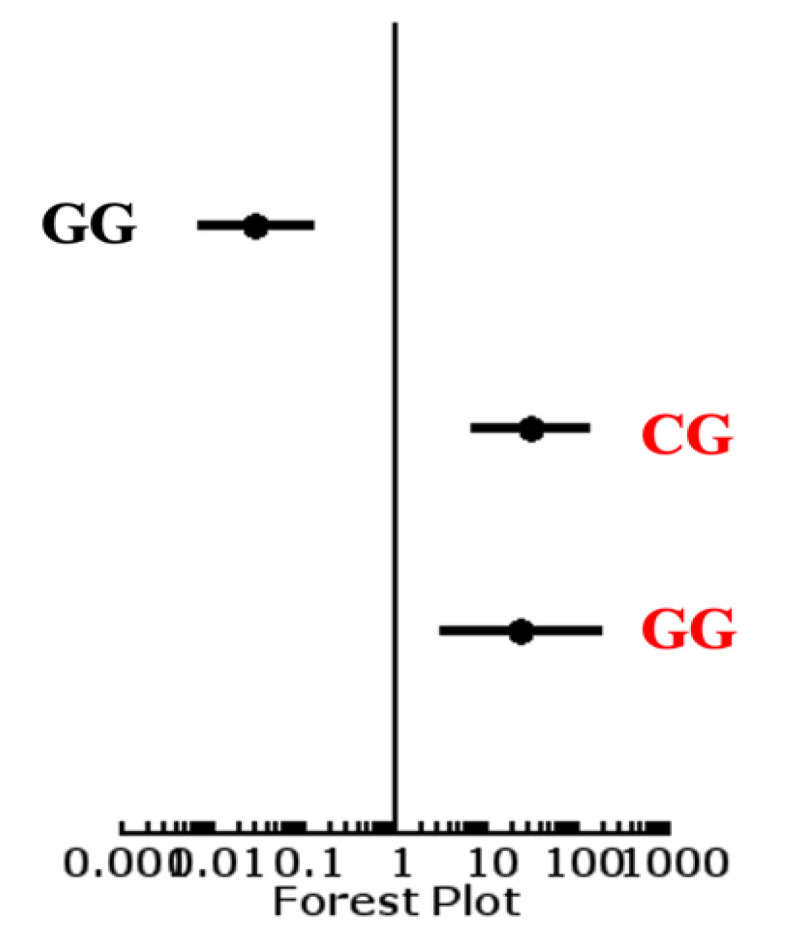
Forest plot of genes of *rs2910164*.

Odds ratios were calculated for the genotypes of *rs2910164*. The odds ratio for homozygous dominant genotype GG was 0.0306 (0.0071–0.13131) with a *p*-value < 0.0001 (<0.05). The odds ratio for heterozygous CG genotype was 30.6748 (7.1467–131.6616) with a *p*-value < 0.0001 at a 95% confidence interval. The odds ratio for the homozygous recessive genotype CC was 3.0833 (1.2649–7.5157) with the *p*-value of 0.010255 at 95% CI (Figure 1).

**Figure 2:**
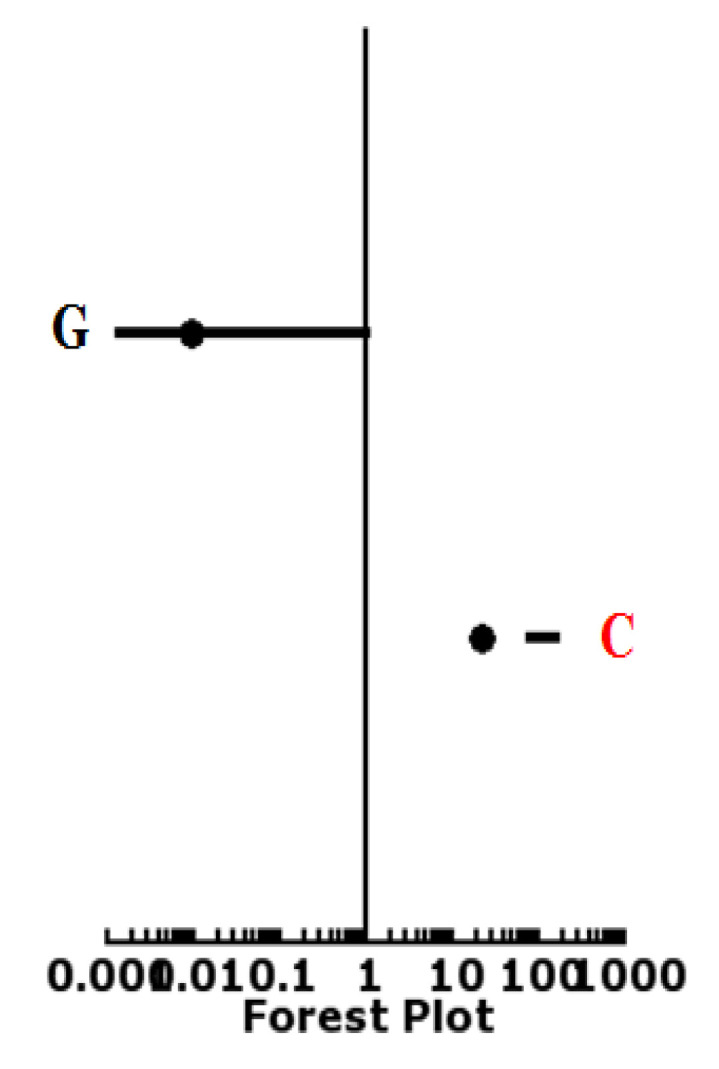
Forest plot of alleles of *rs2910164*.

The odds ratio for alleles of rs2910164 showed a value for the wild type allele G in patients and control of 0.0098 (0.0013–1.073) with a *p*-value < 0.0001; the odds ratio for risk allele C for patients and controls was 23.0168 (3.0321–174.7208) with a *p*-value of <0.0001. These results showed that the frequency of the major allele G was lower in patients while the frequency of minor allele C was higher in patients (Table 4, Figure 2).

**Figure 3:**
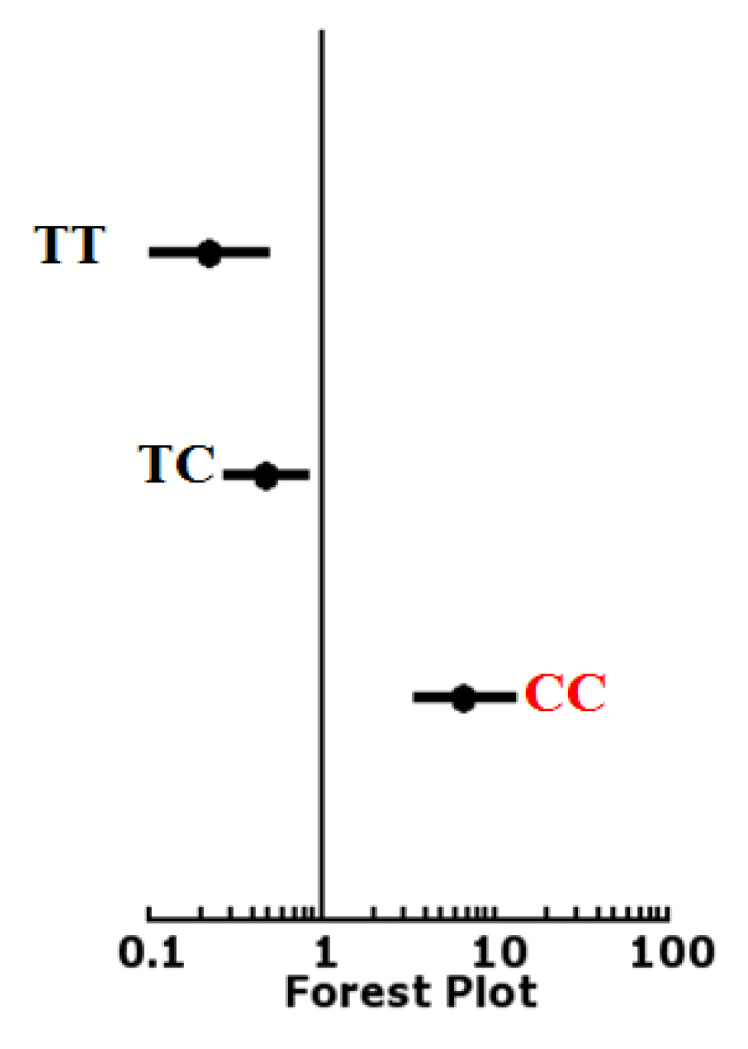
Odd ratio calculation for genotypes of *rs4938723*.

Odds ratios for the patients and control groups were calculated for the genotypes of *rs4938723* in the current study. The odds ratio for homozygous dominant genotype TT was 0.2222 (0.1012–0.488) with a *p*-value < 0.0001 (<0.05). The odds ratio for heterozygous CG genotype were 0.4792 (0.2718–0.8447) with *p*-value < 0.010488 at 95% confidence interval. The odds ratio for the homozygous recessive genotype CC was 6.6522 (3.3862–13.0683) with the *p*-value < 0.0001 at 95% CI. Forest plot also showed that the genotype CC is associated with the disease group (Figure 3).

**Figure 4:**
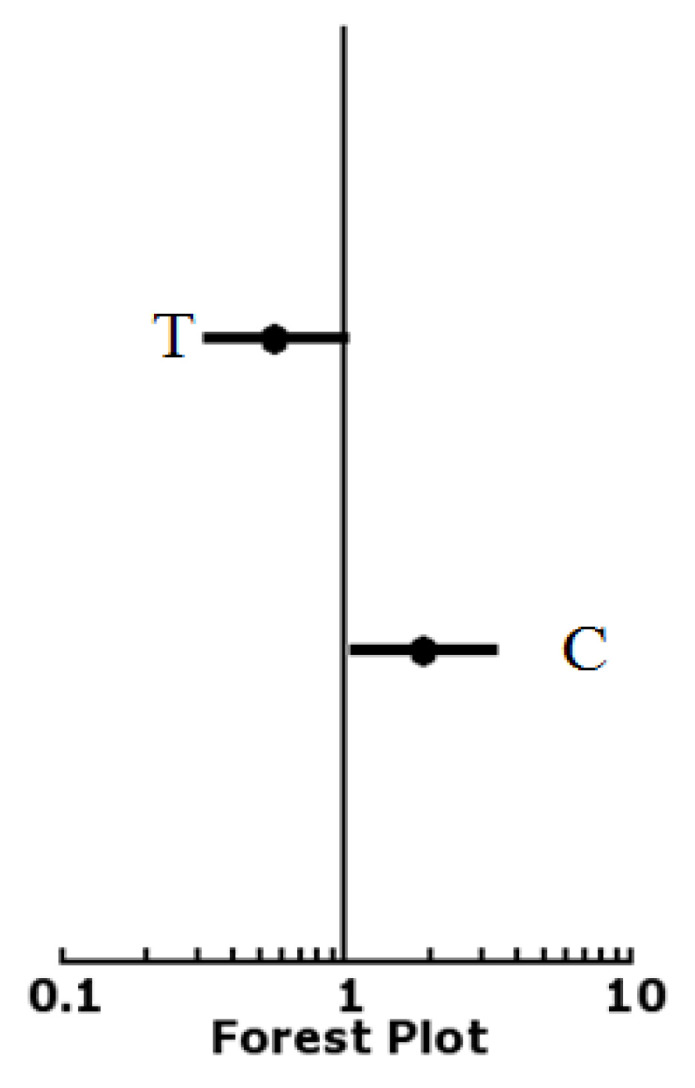
Odd ratio calculation of genotypes of *rs4938723*.

Odds ratios for alleles of *rs4938723* showed that the odds ratio for the wild type allele T in patients and control was 0.5641 (0.3137–1.0142) and *p*-value was <0.05474, while the odds ratio for risk allele C was 1.8621 (1.0321–3.3596) with a *p*-value of <0.03788. These results showed that the frequency of the major allele T was lower in patients while the frequency of minor allele C was higher in patients (Table 4, Figure 4).

#### 3.2.3. Allelic OR Correlation Frequencies of *miRNA-146a rs2910164* and *miRNA-34b/c rs4938723*

Allele frequencies for rs2910164 showed that the frequency of dominant allele G was 49.25% and 99% and the frequency of mutant allele C was 20.75% and 1.0 % in patients and controls, respectively, with the *p*-value 4 × 10^−8^ (<0.05) and a Chi-square was 30.178 showing a statistically significant and strong association of the mutant allele with thyroid cancer risk in the patient group as compared to normal controls (Table 5).

Allele frequencies for *rs4938723* showed that the frequency of dominant allele T was 49.25% and 99% and the frequency of mutant allele C was 20.75% and 1.0% in patients and controls, respectively, with a *p*-value of 0.04765 (<0.05) and a Chi-square value of 3.922. There was a statistically significant and strong association of the mutant allele with thyroid cancer risk in the patient group as compared to normal controls (Table 5).

## 4. Discussion

Jazdzewski et al. found that different polymorphisms affect the expression of miRNA and, in turn, this affects the mRNA maturation or recognition which may be an important risk factor in the susceptibility to disease. A variant was described in the passenger strand of the pre-miRNA (*rs2910164*) of *miRNA-146a*, which is associated with the risk of papillary thyroid cancer and also affects *miRNA-146a* processing. It was found that heterozygous genotype GC is associated with a high risk of papillary thyroid carcinoma while homozygous GG or CC genotypes have not shown any association when cancerous tissues were compared with normal tissues [9]. Chatzikyriakidou et al. found that G > C nucleotide replacement is present in common hsa-mir-146a polymorphism, *rs2910164*, which causes the change from a G:U pair to a C:U mismatch in the basic structure of the mir-146a precursor. This substitution changes the specific activities of mature hsa-mir-146a while binding to its specific targets, which results in increased expression of hsa-mir-146a [21]. Moreover, MITF (Microphthalmia-associated Transcription Factor), is also targeted by *miRNA-146a*. MITF is a factor that is a protooncogenic transcription factor and it acts as a master regulator in the development of melanocyte functioning and survival. It was also implicated in choroidal melanoma pigmentation and proliferation [22]. A study performed by Xiang et al. indicated the existence of the variant C allele of miR-146a correlated with increased risk of squamous cell carcinoma (SCC) and head and neck carcinoma [23]. Data from a study by Peng et al. demonstrated that the CC and CT genotype of *rs493872* significantly increased the risk of PTC [24]. Granja et al. evaluated that the CC genotype frequency is significantly raised in Brazilian patients diagnosed with different types of thyroid carcinoma [25]. Similar results were observed in Russian and Ukrainian populations [26]. A further meta-analysis supported the result that CC genotype is a risk factor for thyroid cancer [16]. The same results were observed in the present study as it was noted that the frequency of the CC genotype was significantly higher in the group of patients with PTC than in controls in the Pakistani population. This shows that the mutations in SNP of *miRNA-34b/c* play a significant role in the development of thyroid cancer.

## 5. Conclusions

Statistical analysis in the present study indicated for the first time that the genetic variability of the *miRNA-146a rs2910164* and *miR-34b/c rs4938723* is associated with the risk of thyroid cancer in the studied population. Further, these studies also indicated that these miRNAs can be used for prognostic and diagnostic purposes in future for the early detection and treatment of thyroid cancer patients.

## Figures and Tables

**Table 1:** Clinical assessment of Thyroid Cancer Patients.

Variables	Patients (*n* = 200)	Frequency (%)	Hormonal Levels		
			TSH	TG	ATG
Age	<50	34	-	-	-
	50–60	52	-	-	-
	>60	14	-	-	-
Gender	Females	86	-	-	-
	Males	14	-	-	-
Metastasis	Metastatic	7	-	-	-
	Non-metastatic	93	-	-	-
Types of cancer	Papillary thyroid cancer	53	42.55	228	81.7
	Follicular variant of PTC	42	42.0	156	232.34
	Hurthle cell cancer	3.0	72.17	132.34	239.65
	Insular papillary thyroid cancer	1.0	64.76	12.96	24.3
	Classical variant of PTC	2.0	55.8	80.3	173.4

**Table 2:** Genotype Frequency Distribution of *rs2910164* and *rs4938723* with the Risk of Thyroid Cancer in Pakistani Population.

Genotypes	Patients Observed Frequency, %	Expected H-W Frequency, %	Control Observed Frequency, %	Expected H-W Frequency, %	*p*-Value for the Risk Assessment between Patients & Controls
**rs2910164**	-	-	-	-	0.00001 **
**GG**	60	62.8056	98	98.01	
**CG**	38.50	32.8888	02	1.98	
**CC**	1.50	4.3056	00	0.01	
	*p*-value = 0.2332	-	*p*-value = 0.99048	-	
**rs4938723**	-	-	*-*	-	2 × 10^−8^ **
**TT**	10	8.12	30.00	32.81	
**TC**	36	40.75	54.50	48.36	
**CC**	54	51.1	15.50	17.81	
	*p*-value = 0.6541	-	*p*-value = 0.51053	-	

** highly significant.

**Table 3:** Risk assessment of rs2910164 and rs4938723 with thyroid cancer and their combined genotype frequency calculations.

Genotypes	Patients Observed Frequency, %	Control Observed Frequency, %	*p*-Value for the Risk Assessment between Patients & Controls
**rs2910164**			
GG	60	98	*p*-value ≤ 0.00001
CG/CC	40	2	Chi-square = 43.52
**rs4938723**			
TT	10	30	*p*-value = 0.00030426
TT/CC	90	70	Chi square = 13.044

**Table 4:** Genotype OR correlation calculations of *rs2910164* and *rs4938723* for risk assessment.

Genotypes	Patients Observed Frequency %	Control Observed Frequency %	Correlation by OR (95%CI) between Patient & Control	*p*-Value for the Risk Assessment between Patients & Controls
rs2910164				
**GG**	60	98	0.03 (0.007–0.13)	<0.0001
**CG**	38.50	02	30.67(7.14–131.66)	<0.0001
**CC**	1.50	00	24.75(3.25–188.43)	<0.0001
rs4938723				
**TT**	10	30.00	0.22(0.10–0.48)	<0.0001
**TC**	36	54.50	0.4792(0.27–0.84)	0.01048
**CC**	54	15.50	6.65(3.38–13.06)	<0.0001

**Table 5:** Allelic OR correlation calculations of *rs2910164* and *rs4938723* for risk assessment.

Alleles	Patients Observed Frequency %	Control Observed Frequency %	Correlation by OR (95%CI) between Patient & Control	*p*-Value for the Risk Assessment between Patients & Controls
**rs2910164**				
G	79.25	99	0.04 (0.005–0.30)	<0.0001
C	20.75	1	23.01 (3.03–174.72)	<0.0001
**rs4938723**				
T	28.57	42.30	0.56 (0.31–1.01)	0.05474
C	71.43	57.70	1.93 (1.07- 3.49)	0.026702

## Data Availability

The material described is not under publication or consideration for publication elsewhere.

## References

[1] Heidari Z., Mohammadpour-Gharehbagh A., Eskandari M., Harati-Sadegh M., Salimi S. (2019). Genetic polymorphisms of miRNA let7a-2 and pri-mir-34b/c are associated with an increased risk of papillary thyroid carcinoma and clinical/pathological features. J. Cell. Biochem..

[2] Torre L.A., Siegel R.L., Ward E.M., Jemal A. (2016). Global cancer incidence and mortality rates and trends—An update. Cancer Epidemiol. Prev. Biomark..

[3] Li X., Abdel-Mageed A.B., Mondal D., Kandil E. (2013). MicroRNA expression profiles in differentiated thyroid cancer, a review. Int. J. Clin. Exp. Med..

[4] Suresh R., Sethi S., Ali S., Giorgadze T., Sarkar F.H. (2015). Differential expression of MicroRNAs in papillary thyroid carcinoma and their role in racial disparity. J. Cancer Sci. Ther..

[5] Labbaye C., Testa U. (2012). The emerging role of MIR-146A in the control of hematopoiesis, immune function and cancer. J. Hematol. Oncol..

[6] Budhu A., Ji J., Wang X.W. (2010). The clinical potential of microRNAs. J. Hematol. Oncol..

[7] Zhong H., Wang H.-r., Yang S., Zhong J.-h., Wang T., Wang C., Chen F.-y. (2010). Targeting Smad4 links microRNA-146a to the TGF-β pathway during retinoid acid induction in acute promyelocytic leukemia cell line. Int. J. Hematology..

[8] Jazdzewski K., Liyanarachchi S., Swierniak M., Pachucki J., Ringel M.D., Jarzab B., De la Chapelle A. (2009). Polymorphic mature microRNAs from passenger strand of pre-miR-146a contribute to thyroid cancer. Proc. Natl. Acad. Sci. USA.

[9] Jazdzewski K., Murray E.L., Franssila K., Jarzab B., Schoenberg D.R., de la Chapelle A. (2008). Common SNP in pre-miR-146a decreases mature miR expression and predisposes to papillary thyroid carcinoma. Proc. Natl. Acad. Sci. USA.

[10] Jeon H.-S., Lee Y.H., Lee S.Y., Jang J.-A., Choi Y.-Y., Yoo S.S., Lee W.K., Choi J.E., Son J.W., Kang Y.M. (2014). A common polymorphism in pre-microRNA-146a is associated with lung cancer risk in a Korean population. Gene.

[11] Marino M., Cirello V., Gnarini V., Colombo C., Pignatti E., Casarini L., Diazzi C., Rochira V., Cioni K., Madeo B. (2013). Are pre-miR-146a and PTTG1 associated with papillary thyroid cancer?. Endocr. Connect..

[12] Khan R., Shaheen H., Mansoor Q., Abbasi S.A., Fatima S., Ammar A., Baig R.M. (2021). Genetic predisposition of SNPs in miRNA-149 (rs2292832) and FOXE1 (rs3758249) in thyroid Cancer. Mol. Biol. Rep..

[13] Xu Y., Liu L., Liu J., Zhang Y., Zhu J., Chen J., Liu S., Liu Z., Shi H., Shen H. (2011). A potentially functional polymorphism in the promoter region of miR-34b/c is associated with an increased risk for primary hepatocellular carcinoma. Int. J. Cancer.

[14] Bartel D.P. (2004). MicroRNAs: Genomics, biogenesis, mechanism, and function. Cell.

[15] Botchkarev V.A., Flores E.R. (2014). p53/p63/p73 in the epidermis in health and disease. Cold Spring Harb. Perspect. Med..

[16] Wu B., Guo D., Guo Y. (2014). Association between p53 Arg72Pro polymorphism and thyroid cancer risk: A meta-analysis. Tumor Biol..

[17] Son M.S., Jang M.J., Jeon Y.J., Kim W.H., Kwon C.-I., Ko K.H., Park P.W., Hong S.P., Rim K.S., Kwon S.W. (2013). Promoter polymorphisms of pri-miR-34b/c are associated with hepatocellular carcinoma. Gene.

[18] Hashemi M., Danesh H., Bizhani F., Narouie B., Sotoudeh M., Nouralizadeh A., Sharifiaghdas F., Bahari G., Taheri M. (2017). Pri-miR-34b/c rs4938723 polymorphism increased the risk of prostate cancer. Cancer Biomark..

[19] Yi D.-H., Wang B.-G., Zhong X.-P., Liu H., Liu Y.-F. (2014). Pri-miR-34b/c rs4938723 TC heterozygote is associated with increased cancer risks: Evidence from published data. Tumor Biol..

[20] Gao L.-B., Li L.-J., Pan X.-M., Li Z.-H., Liang W.-B., Bai P., Zhu Y.-H., Zhang L. (2013). A genetic variant in the promoter region of miR-34b/c is associated with a reduced risk of colorectal cancer. Biol. Chem..

[21] Chatzikyriakidou A., Voulgari P.V., Georgiou I., Drosos A.A. (2012). miRNAs and related polymorphisms in rheumatoid arthritis susceptibility. Autoimmun. Rev..

[22] Yajima M., Miyata M., Ikuta K., Hasegawa Y., Oneyama C., Kanda T. (2019). Efficient Epstein-Barr virus progeny production mediated by cancer-derived LMP1 and virally-encoded microRNAs. Microorganisms.

[23] Xiang Z., Song J., Zhuo X., Li Q., Zhang X. (2017). MiR-146a rs2910164 polymorphism and head and neck carcinoma risk: A meta-analysis based on 10 case-control studies. Oncotarget.

[24] Chen P., Sun R., Pu Y., Bai P., Yuan F., Liang Y., Zhou B., Wang Y., Sun Y., Zhu J. (2015). Pri-miR-34b/C and Tp-53 polymorphisms are associated with the susceptibility of papillary thyroid carcinoma: A case–control study. Medicine.

[25] Granja F., Morari J., Morari E.C., Correa L.A., Assumpção L.V., Ward L.S. (2004). Proline homozygosity in codon 72 of p53 is a factor of susceptibility for thyroid cancer. Cancer Lett..

[26] Rogounovitch T.I., Saenko V.A., Ashizawa K., Sedliarou I.A., Namba H., Abrosimov A.Y., Lushnikov E.F., Roumiantsev P.O., Konova M.V., Petoukhova N.S. (2006). TP53 codon 72 polymorphism in radiation-associated human papillary thyroid cancer. Oncol. Rep..

